# Rehabilitation nach Nierentransplantation: Stationäre multimodale Rehabilitation als Bestandteil der (Langzeit‑)Nachsorge nach Nierentransplantation und nach Nierenlebendspende

**DOI:** 10.1007/s11560-021-00484-4

**Published:** 2021-02-08

**Authors:** Doris Gerbig

**Affiliations:** Fachklinik Bad Heilbrunn, Innere Medizin – Nephrologie/Transplantationsnachsorge, m&i - Fachklinik Bad Heilbrunn, Wörnerweg 30, 83670 Bad Heilbrunn, Deutschland

**Keywords:** Nieren, Transplantatüberleben, Lebendspende, Rehabilitation, Nachsorge, Kidneys, Transplant survival, Living donation, Rehabilitation, Aftercare

## Abstract

**Hintergrund:**

Durch strukturierte (Langzeit‑)Nachsorge nach Nierentransplantation und Nierenlebendspende können Risikofaktoren für Transplantatüberleben und Nierenfunktion und für die physische wie psychische Morbidität unterschiedlichster Genese detektiert und gebessert werden. Neben den klassischen kardiovaskulären Risikofaktoren zählen hierzu eine mangelnde Adhärenz des Patienten, Wissensdefizite im Hinblick auf Verhaltensregeln nach Transplantation und Lebendspende, Bewegungsmangel, mangelnde Coping-Strategien oder auch arbeitsmedizinische und sozialrechtliche Belange.

**Ziel der Arbeit:**

Es soll dargestellt werden, wie Rehabilitationsmaßnahmen die Nachsorge nach Nierentransplantation und Lebendspende optimieren können, worauf sich der Rehabilitationsbedarf begründet, welche Ziele verfolgt werden und welche multidisziplinären Therapiemodule sich etabliert haben.

**Material und Methoden:**

Hierfür dienen neben einer Literaturrecherche die Erfahrungen einer Rehabilitationsklinik, die seit dem Jahr 2000 Rehabilitationen nach Nierentransplantation und nach Lebendspende anbietet und etwa 600 Patienten pro Jahr behandelt.

**Ergebnisse:**

Spezialisierte Rehamaßnahmen mit nephrologischem und transplantationsmedizinischem Schwerpunkt können die ambulante Nachsorge nach Nierentransplantation und Nierenlebendspende sinnvoll ergänzen, wenn die Rehabilitationsklinik konzeptionelle, personelle und strukturelle Qualitätsanforderungen erfüllt. Eine enge Kooperation der Rehaklinik mit dem Transplantationszentrum und den behandelnden Nephrologen ist essenziell.

**Diskussion:**

Kontrollierte Studien zur Langzeitnachsorge unter Einbeziehung der stationären Rehabilitation sind anzustreben. Auch Prärehabilitation sollte in diesen Kontext einbezogen werden.

## Abstract (en)

**Background:**

Structured follow-up care starting immediately after the transplantation and living donation and ideally continuing long-term, can improve transplant survival and aid in the detection of risk factors for physical and psychological morbidity of various origins. In addition to classical cardiovascular risk factors, these include a lack of patient adherence, knowledge deficits with respect to the rules of conduct after transplantation and living donation, lack of exercise and coping strategies and also occupational health and social law issues.

**Objective:**

This article aims to clarify how rehabilitation measures can optimize the aftercare following kidney transplantation and living donation, what the need for rehabilitation is based on, what goals are pursued and which multidisciplinary therapy modules have been established.

**Material and methods:**

For this purpose, the experiences of a rehabilitation clinic offering rehabilitation after kidney transplantation and living donation and currently treats approximately 600 patients per year since 2000, have been considered in addition to a literature search.

**Results:**

Given the complexity surrounding posttransplantation aftercare, specialized rehabilitation measures focusing on nephrology and transplantation medicine can meaningfully supplement outpatient aftercare after kidney transplantation and living donation, if the rehabilitation clinic fulfils conceptual, personnel, and structural quality requirements. A close cooperation between the rehabilitation clinic, the transplantation center, and the treating nephrologist is essential.

**Conclusion:**

Controlled studies on the effects of long-term aftercare including inpatient rehabilitation are still needed. In this context, prerehabilitation should also be included.

Zu einem guten Verlauf nach Transplantation und Lebendspende trägt die Nachsorge wesentlich bei. Patienten nach Nierentransplantation müssen im Umgang mit der neuen Situation nach oft langjähriger Dialysepflichtigkeit sicher sein. Dies betrifft nicht nur das medizinische Umfeld mit dem Fokus Immunsuppression und deren Nebenwirkungen sowie renale Risikofaktoren, sondern auch Komorbiditäten, allgemein die physische und psychische Verfassung der Patienten sowie ihre arbeitsmedizinischen und sozialrechtlichen Belange. Auch Lebendnierenspender haben einen klaren Nachsorgebedarf im Hinblick auf die Thematik Nierenschutz, die psychische Situation nach der Spende und auf die neue Beziehung zum Nierenempfänger, der für den Nierenspender einen wesentlichen Kontextfaktor darstellt. Da das ambulante Setting oft nicht für die Vertiefung dieser vielfältigen Themen ausreicht, kann eine spezialisierte multimodale stationäre Rehabilitationsmaßnahme die Nachsorge sinnvoll ergänzen.

## Transplantation – eine Herausforderung für Vor- und Nachsorge

Die Nierentransplantation hat sich zu einem erfolgreichen Nierenersatzverfahren entwickelt, das Patienten mit terminalem Nierenversagen ein nahezu normales Leben ermöglichen kann, wenn die medizinischen, psychologischen und sozialen Rahmenbedingungen gut eingestellt sind. Im Vergleich zur Dialyse bedeutet Transplantation für die Patienten eine höhere Lebenserwartung, weniger renale Folgeerkrankungen und eine geringere Inzidenz von Komorbiditäten [28]. Bemerkenswert ist, dass der Vorteil einer Transplantation mit zunehmendem Alter steigt [29].

Transplantation kann ein nahezu normales Leben ermöglichen

Terminal niereninsuffiziente Patienten sehen sich in Deutschland mit einer oft jahrelangen Wartezeit auf ein postmortal gespendetes Organ konfrontiert [11]. Nehmen auf der Warteliste Morbidität, Inaktivität und Gebrechlichkeit („frailty“; [20]) während der langjährigen Dialysetherapie zu, kann der Erfolg einer Transplantation gefährdet sein [30]. Frailty-Kriterien auf der Warteliste oder nach Transplantation gehen mit einer verschlechterten Transplantatfunktion, einer erhöhten Mortalität und häufigeren stationären Aufenthalten nach Transplantation einher, wenn man diese Patienten mit Patienten vergleicht, die in einem „guten Zustand“ transplantiert werden und diesen auch nach Transplantation erhalten [17, 20].

In diesem Kontext haben Nierenlebendspenden [19] eine besondere Bedeutung: Durch eine Lebendspende, die idealerweise präemptiv oder zu Beginn der Dialysepflicht erfolgt [1], kann man einem nahestehenden Menschen viele Jahre Wartezeit auf ein Organ ersparen und so wesentlich zum Transplantationserfolg beitragen.

Sowohl transplantierte Patienten als auch Lebendspender sind Patientengruppen, die einen besonderen Nachsorgebedarf aufweisen [17, 18] – beginnend von medizinisch hochwertiger Versorgung über krankheitsspezifische Schulungen, psychologische Leistungen, angepasste körperliche Aktivität bis hin zu sozialrechtlichen und arbeitsmedizinischen Belangen. In der Regel kann das ambulante Setting der Nachsorge diese Bereiche nicht vollumfänglich abdecken. Eine multimodale und interdisziplinäre Rehabilitation in einer spezialisierten nephrologischen Rehaklinik kann die ambulante Nachsorge direkt nach den operativen Eingriffen und im Langzeitverlauf sinnvoll ergänzen.

## Bedeutung der Rehabilitation nach Transplantation (Rehabedarf, Rehafähigkeit)

Grundsätzlich müssen nach den Richtlinien des Gemeinsamen Bundesausschusses (G-BA) über Leistungen zur medizinischen Rehabilitation [13] auf der Grundlage des §111a Sozialgesetzbuch Fünftes Buch (SGB V) und der Internationalen Klassifikation der Funktionsfähigkeit, Behinderung und Gesundheit (ICF; [9]) vor Genehmigung einer Rehabilitationsmaßnahme individuell Rehabilitationsbedürftigkeit, -fähigkeit, -prognose und -ziele definiert werden.

Bei chronischen Erkrankungen wie der chronischen Niereninsuffizienz sollen durch Rehabilitation körperliche, berufliche und soziale Fähigkeiten erhalten oder wiedererlangt werden. Nach Transplantation können die vielschichtigen Einschränkungen nach der Dialysetherapie noch in unterschiedlichem Ausmaß weiterbestehen. Zudem sieht sich der transplantierte Patient mit einer Gemengelage konfrontiert, bei deren Klärung er intensive medizinische, psychologische, sozialberatende und physiotherapeutische Unterstützung benötigt und einen hohen Informationsbedarf hat. Den Langzeitverlauf beeinträchtigende Kontextfaktoren müssen ebenfalls berücksichtigt und ggf. verbessert werden.

Aus all diesen vielschichtigen Beeinträchtigungen kann der Rehabilitationsbedarf für den transplantierten Patienten abgeleitet werden, da „über die kurative Versorgung hinaus der mehrdimensionale und interdisziplinäre Ansatz der medizinischen Rehabilitation erforderlich ist“ [13].

Voraussetzung für eine Rehabilitation sind darüber hinaus die Rehabilitationsfähigkeit (ausreichende physische und psychische Belastbarkeit, die es erlaubt, dass der Patient an der Rehabilitationsmaßnahme auch teilnehmen kann), eine entsprechende Motivation und eine positive Rehabilitationsprognose. Letztere ist nach Transplantation als sehr hoch einzustufen, da die Patienten nach erfolgreicher Transplantation hoch motiviert sind, alles dafür zu tun, damit das Transplantat möglichst lange eine möglichst gute Funktion behält und somit eine erneute Dialysepflichtigkeit vermieden bzw. verzögert wird.

## Ziele einer Rehabilitation nach Transplantation

Durch Rehamaßnahmen sollen möglichst frühzeitig alltagsrelevante Beeinträchtigungen der Aktivitäten beseitigt oder vermindert werden, eine Verschlimmerung verhütet oder eine drohende Beeinträchtigung abgewendet werden. Weiter sollen die klinischen Langzeitergebnisse verbessert, die physische und psychische Lebensqualität der Patienten gesteigert und so eine bessere Teilhabe ermöglicht werden.

In Infobox 1 werden Ziele der medizinischen Rehabilitation von transplantierten Patienten in Anlehnung an die Leitlinien zur kardiologischen Rehabilitation [14] zusammengefasst.

### Infobox 1 Ziele der Rehabilitation nach Nierentransplantation [14]

- *Verbesserung der Lebensqualität:*
  - Reduktion der Beschwerden, die mit chronischer Nierenerkrankung und nach Transplantation einhergehen (z. B. Polyneuropathie, Anämie, Osteopathie, Nebenwirkungen der Immunsuppressiva u. v. m.; [28])
  - Verbesserung der körperlichen Funktion und Leistungsfähigkeit [5, 6, 15, 25]
  - Verhinderung eines Gebrechlichkeitssyndroms („frailty“; [20])
  - Stabilisierung des psychischen Befindens (Krankheitsbewältigung, Organakzeptanz, Umgang mit der Erkrankung im Alltag, bei Lebendspende Berücksichtigung des Kontextfaktors „Spender“; [17])
  - Ermöglichung und Gewährleistung der sozialen Wiedereingliederung und Teilhabe (leidensgerechte Berufstätigkeit, Familie, Erhaltung der Selbständigkeit bei alten Patienten)
- *Verbesserung der Prognose *[17]:
  - Prävention und Risikoreduktion
  - Reduktion der Morbidität
  - Reduktion der Mortalität
- *Beitrag zur Kostenstabilität:*
  - Verbesserung der Adhärenz [23, 27]
  - Reduktion/Verhinderung vermeidbarer Krankenhausaufenthalte [20]
  - Verhinderung einer erneuten Dialysetherapie
  - Vermeidung von vorzeitiger Berentung und Pflege

## Ansatzpunkte der Reha: Faktoren, die das Outcome einer Transplantation beeinflussen

Das Erkennen und die Behandlung von Risikofaktoren und negativen Kontextfaktoren ist wesentlich für das Langzeitüberleben des Transplantats [17]. Zu den Faktoren, die die Transplantatfunktion verschlechtern können, zählen unter anderem arterielle Hypertonie, Diabetes mellitus, hier insbesondere der Postransplantationsdiabetes mellitus (PTDM; [2]), Hyperlipoproteinämie, Adipositas, aber auch virale und bakterielle Infektionen sowie Neoplasien. Diese Sekundärerkrankungen bzw. Komplikationen können zum Teil auch als Nebenwirkungen der Immunsuppressiva auftreten.

Auch eine unzureichende Therapieadhärenz kann bei Patienten aller Altersgruppen die Transplantatfunktion gefährden [17, 27]. Die Adhärenzproblematik wird von Patient und Behandler im ambulanten Setting möglicherweise unterschiedlich eingeschätzt [23].

Zum Gelingen der Transplantation trägt weiter eine psychische Stabilität des Patienten bei. Mangelnde Organakzeptanz, Angst vor erneuter Dialysepflichtigkeit, Überforderung oder auch ein neu zu adjustierendes Verhältnis zum Lebendnierenspender sind nur einige Themen, mit denen sich der Patient nach der Transplantation unter Umständen konfrontiert sieht. Auch muss der Patient nach einer Transplantation die Motivation finden, den Risikofaktor Rauchen zu überwinden [17].

Körperliche Aktivität ist nicht nur bei chronischer Nierenerkrankung, sondern auch nach Transplantation von besonderer Bedeutung [5, 15, 20, 25]. Positive Effekte moderaten Ausdauertrainings sind im Hinblick auf die Senkung des Herz-Kreislauf-Risikos und die verbesserte Pumpfunktion des Herzens beschrieben. Weiterhin können alltagsrelevante körperliche Funktionen gebessert und die Entwicklung von Muskelabbau und „frailty“ verringert werden. Insgesamt lassen sich bei nierenkranken Patienten eine Verminderung der Depressivität und eine deutliche Steigerung der Lebensqualität erreichen. Es besteht ein klarer Zusammenhang zwischen Mortalität nach Transplantation und körperlicher Aktivität zum Zeitpunkt der Transplantation. Empfohlen wird, ein moderates Ausdauertraining mit 5‑mal 30 min pro Woche regelmäßig durchzuführen [16]. Chronisch niereninsuffizienten Patienten fällt es jedoch schwer, einen Zugang zu regelmäßiger körperlicher Aktivität zu finden [6, 15].

Schließlich sollen vom Patienten spezielle Hygiene- und Ernährungsempfehlungen vor dem Hintergrund der Immunsuppression berücksichtigt und die Ernährung und Trinkmengengestaltung auf die „neue Niere“ eingestellt werden.

Berufstätige Patienten müssen prüfen (lassen), ob ihre Tätigkeit nach Transplantation „leidensgerecht“ ist. Erwerbsminderungsrenten und der Grad der Behinderung (GdB) werden evtl. nachjustiert.

### „Trainingscamp“ Reha

Die Vielfältigkeit der Anforderungen, mit denen sich ein Patient nach Transplantation konfrontiert sieht und die er erlernen und beachten muss, kann zu einer Überforderung des Patienten führen und den Erfolg der Transplantation gefährden [6]. Deshalb muss der Patient direkt nach Transplantation, aber auch im Langzeitverlauf professionell unterstützt und beraten werden. Diese besondere Form der Nachsorge kann durch die ambulante Transplantationsnachsorge in Kombination mit einer multimodalen, interdisziplinären Rehamaßnahme in einer spezialisierten Rehaklinik erreicht werden. Die stationäre Rehabilitationsmaßnahme stellt somit ein „Trainingscamp“ für Transplantierte dar.

## Neuland in der Rehabilitation: Reha nach Nierenlebendspende

Auch wenn im Transplantationsgesetz der Anspruch auf Rehabilitation klar verankert ist [4], war für Patienten, Behandler und auch Kostenträger nicht immer ersichtlich, warum ein „gesunder“ Nierenspender an einer Reha teilnehmen sollte.

Aufgrund der außergewöhnlichen Situation eines Spenders [18] ist jedoch ein klarer Rehabilitationsbedarf abzuleiten und Rehaziele nach der Lebendspende sind zu definieren:

- Nierenschutz muss erlernt werden

Dem Spender müssen bei der neu eingetretenen Einnierigkeit die bekannten Empfehlungen zum Nierenschutz nahegebracht werden [18, 22]. Nach der Nierenspende ist es deshalb geboten, dass eine gründliche medizinische Evaluation auch im Langzeitverlauf stattfindet. Detaillierte Informationen und Hilfestellungen zur Gewährleistung eines „nierenschonenden Verhaltens“ können in einer Rehamaßnahme für Nierenspender zusammen mit dem Patienten erarbeitet werden, damit eine nachhaltige Verhaltensänderung beim Spender unter dem Aspekt der Selbstfürsorge initiiert wird.

- psychologische Unterstützung nach der Nierenspende

Bei der Nephrektomie zur Nierenspende handelt es sich nicht um einen Heileingriff, sondern um eine altruistische Tat zum Wohle eines anderen [1]. Vor diesem Hintergrund muss sich der Spender nach der Nierenspende mit einer gezielten Störung der Unversehrtheit des eigenen Körpers auseinandersetzen. Eventuelle Missempfindungen und körperliche Einschränkungen in der ersten Zeit nach der Operation überraschen und erschrecken manche. Mögliche Ängste um die verbliebene Niere kommen erst nach der Prozedur auf, insbesondere wenn der Spender damit gerechnet hat, dass er innerhalb kürzester Zeit wieder das „alte“ physische und auch berufliche Leistungsniveau erreichen würde.

Zudem kann es beim Empfänger postoperativ zu Komplikationen kommen, die den Spender verunsichern [19]. Auch kann es vorkommen, dass sich der Transplantierte mit sehr gut funktionierendem Organ schnell von seinen urämischen Symptomen erholt und es ihm subjektiv vorübergehend sogar besser geht als dem Nierenspender [3].

Bei all diesen Szenarien benötigt der Spender zur Entlastung und Stabilisierung eine hohe Wertschätzung durch erfahrene Ärzte und Therapeuten; er braucht Bestätigung, dass die Entscheidung richtig war.

- Kontextfaktor Empfänger

Die beschriebenen Konstellationen zeigen, wie wichtig die Berücksichtigung des Kontextfaktors Empfänger für den Nierenspender (und umgekehrt) ist, weshalb die Paarvariante eines gemeinsamen Rehabilitationsverfahrens nach unserer Erfahrung besonders zielführend ist.

Die Paarbeziehung Spender/Empfänger war unter Umständen jahrelang von der (Um‑)Sorge des Spenders für den Empfänger geprägt, sei es als Elternteil oder als Lebenspartner. Nach einer erfolgreichen Lebendtransplantation sieht sich der Spender mit einer neuen, ambivalenten Situation konfrontiert: Er empfindet weiterhin große Verantwortung für den Transplantierten, dieser jedoch beginnt die Krankenrolle abzulegen und immer mehr Autonomie zu gewinnen. Psychologische Begleitung von Spender und Empfänger in diesem Stadium hat das Ziel, die Veränderungen in der Paarbeziehung nach der Lebendspende herauszuarbeiten und Lösungswege zur Stabilisierung aufzuzeigen.

## Therapiemodule der stationären Rehabilitation nach Transplantation und Lebendspende am Beispiel der Fachklinik Bad Heilbrunn

Um die oben genannten Ziele zu erreichen, wurden in der Abteilung Nephrologie – Transplantationsnachsorge der Fachklinik Bad Heilbrunn in den letzten 20 Jahren Rehabilitationsmodule speziell für Patienten nach Nierentransplantation und nach Nierenlebendspende entwickelt. Pro Jahr werden etwa 600 Patienten in dieser Abteilung rehabilitiert, davon in etwa 400 nach Nierentransplantation und knapp 100 nach Nierenlebendspende.

Aus folgenden Therapiemodulen setzen sich die Rehamaßnahmen zusammen:

- medizinische Evaluation und Betreuung,
- psychologische Leistungen,
- Physio- und Sporttherapie,
- Schulungen,
- Sozialberatung.

**Figure 1:**
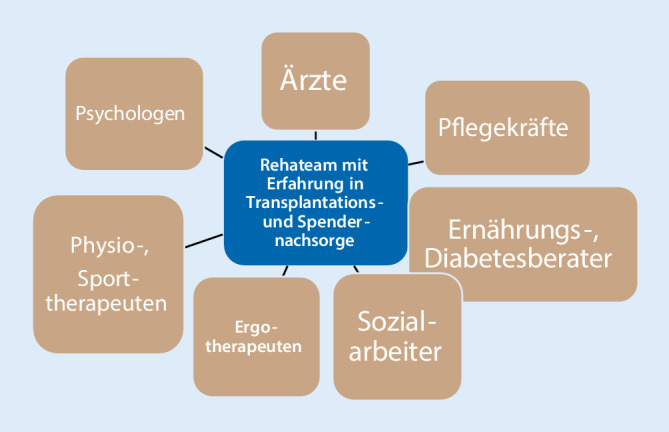

Die Therapiemodule werden vom multiprofessionellen Therapeutenteam (Abb. 1) individuell auf den Patienten sowie abhängig von seinem physischen und psychischen Zustand und von seinen persönlichen Rehazielen angepasst.

Die Rehaklinik muss alle nephrologischen Ver- und Transplantationsnachsorgemöglichkeiten vorhalten

**Figure 2:**
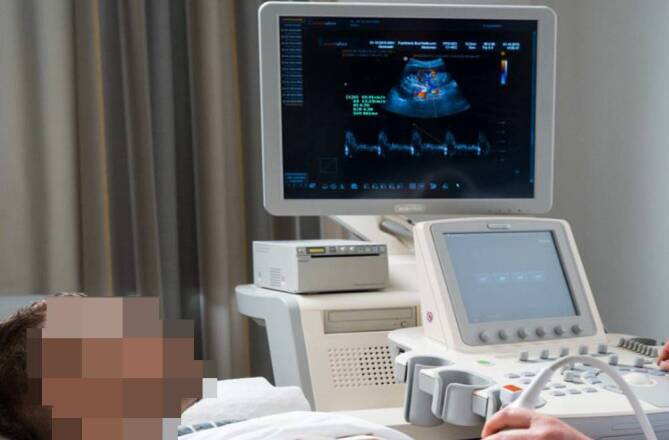

Bei der medizinischen Betreuung ist es essenziell, dass die Rehaklinik alle Möglichkeiten einer nephrologischen Versorgung und Transplantationsnachsorge strukturell und personell inkl. des fachspezifischen Hintergrunds vorhält [14] und sich im Bedarfsfall mit dem einweisenden Zentrum über das weitere Vorgehen abstimmt. Eine breite internistische apparative (z. B. Abb. 2) und Labordiagnostik (inkl. Immunsuppressivaspiegel) sowie eine Dialysemöglichkeit müssen vorhanden sein. Die tägliche Arztvisite und das kontinuierliche diagnostische Monitoring sind Voraussetzungen für eine adäquate medizinische Versorgung.

**Figure 3:**
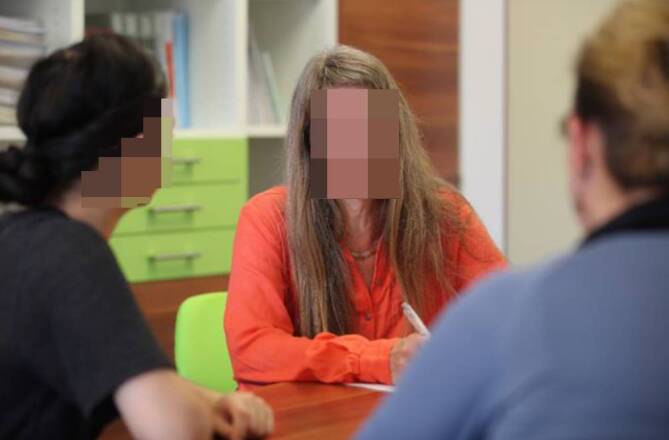

Es soll durch intensive psychologische Leistungen in Einzel- und ggf. (nach Lebendspende) Paargesprächen (Abb. 3) und auch Gesprächsgruppen eine Einschätzung des subjektiven Krankheits- bzw. Transplantationserlebens sowie der individuellen Bewältigungsmechanismen und Ressourcen der Patienten erfolgen. Falls eine weitere psychotherapeutische ambulante Behandlung ratsam erscheint, wird dies mit den Patienten diskutiert.

Die integrativen psychologischen Leistungen basieren auf Methoden der kognitiven Verhaltenstherapie, der Gesprächstherapie und der systemischen Therapie.

Krankheitsspezifische Schulungen in Form von Vorträgen und Einzelschulungen informieren die Patienten ausführlich über ihre Erkrankung, renale Risikofaktoren und Nierenschutz, die Nebenwirkungen der Immunsuppression, Hygieneempfehlungen und eine angepasste Ernährung. Patienten mit PTDM profitieren von spezieller Diabetesberatung und -schulung [2]. Während einer mehrwöchigen Rehabilitation kann der Patient gerade in Bezug auf Adhärenz und Therapieverständnis individuell geschult und der Erfolg der Schulungen überprüft werden [22].

**Figure 4:**
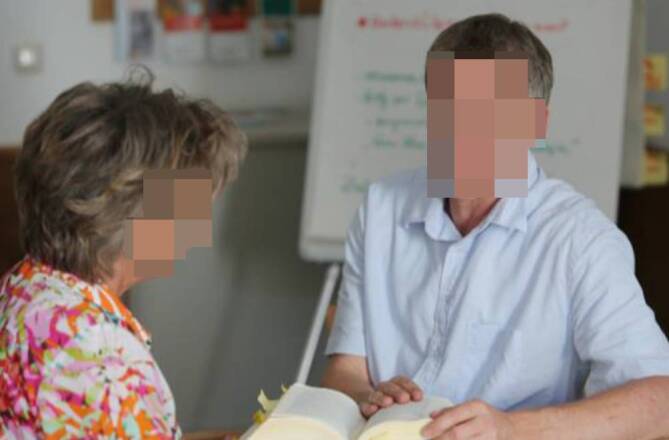

Die vielfältigen Themen bei der Sozialberatung umfassen u. a. Stellungnahmen zur Arbeitsfähigkeit, zu leidensgerechter Tätigkeit, die Beratung bei beruflicher Wiedereingliederung, Umschulung, Erwerbsminderung und anstehender Berentung oder auch die Beratung zum Nachteilsausgleich (Abb. 4). Neben sozialrechtlichen Ansprüchen werden auch die Handhabung von Alltagsproblemen und der Erhalt der Selbstständigkeit thematisiert oder es wird – falls notwendig – eine nachstationäre Versorgung vorbereitet.

Vor dem Hintergrund eines kognitiv-verhaltenstherapeutischen Ansatzes ist die Physio- und Sporttherapie ein wichtiger integraler Baustein, der die psychoedukative Behandlung handlungsorientiert und auf nonverbaler Ebene ergänzt. Freude an Bewegung oder positives Körpererleben soll mit Hilfe der Physio- und Sporttherapie realisiert werden und somit bei den Patienten eine nachhaltige Verhaltensänderung in Richtung regelmäßiger körperlicher Aktivität auch nach der Reha initiieren.

Hierbei muss der Therapeut die Latenz zu Transplantation und Lebendspende berücksichtigen. Zumindest in den ersten 3 Monaten nach Transplantation soll die Bauch- und Beckenregion geschont werden.

**Figure 5:**
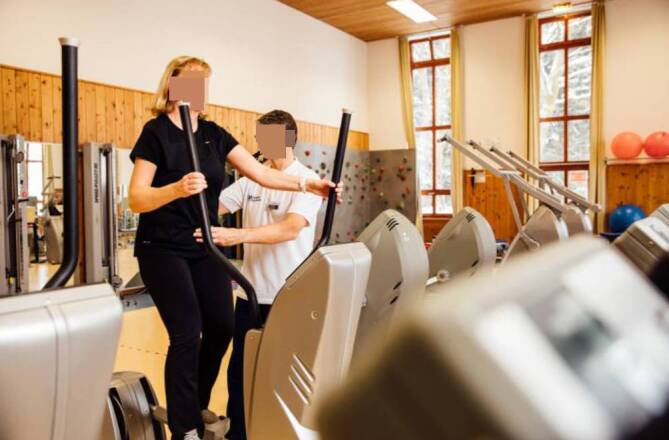

Das sporttherapeutische Angebot umfasst Aufbautraining, Ausdauertraining, Muskelkräftigung und Koordinationstraining. Dies wird beispielsweise durch medizinische Trainingstherapie, Ergometertraining, Durchführung von Bewegungsspielen, Geh- und Lauftraining sowie Übungsgruppen mit unterschiedlichen Schweregraden erreicht (Abb. 5 und 6). Die einzel- und gruppentherapeutischen Übungen werden mit passiven Anwendungen (z. B. Massagen, Wärme- und Stromtherapie) kombiniert.

**Figure 6:**
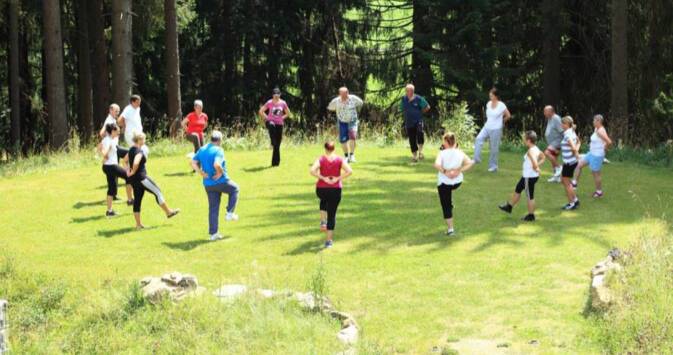

## Diskussion

Rehabilitation ist im SGB IX in das Gesamtkonzept der sozialen Sicherung eingebunden und somit eine gesetzliche Aufgabe. Für viele Indikationen wie beispielsweise nach Hüft-TEP (Totalendoprothese) oder Apoplex ist eine Rehamaßnahme nach dem Ereignis für Patient, Behandler und Kostenträger ein wesentlicher Baustein im Gesundungsprozess. Entsprechend flächendeckend können in Deutschland spezialisierte Rehakliniken für die häufigen medizinischen Indikationen gefunden werden.

In den Indikationen Nephrologie und Nierentransplantation (Indikationsgruppen 8 und 13a nach AHB(Anschlussheilbehandlung)-Indikationskatalog der Deutschen Rentenversicherung (DRV)) ist die Anzahl der spezialisierten Rehakliniken überschaubar, wie eine Recherche der Kommission Rehabilitation der Deutschen Gesellschaft für Nephrologie (DGfN) ergeben hat [10].

Nephrologische Patienten scheinen nicht im Fokus des Gesundheitssystems zu liegen

Woran liegt das? Insgesamt scheinen nephrologische Patienten trotz ihrer schwerwiegenden Erkrankung mit häufigen renalen Folgeerkrankungen und Komorbiditäten nicht im Fokus des Gesundheitssystems zu liegen [12]. Verglichen z. B. mit der Anzahl von Patienten mit Herz-Kreislauf-Erkrankungen in Deutschland (1,7 Mio. Krankenhauseinweisungen in 2017; [8]), nehmen terminal niereninsuffiziente Patienten (ca. 90.000 Dialysepatienten und 23.000 nierentransplantierte Patienten, etwas mehr als 2000 Nierentransplantationen pro Jahr inkl. Lebendspenden) einen eher geringen Raum ein [11, 21].

Dann muss eine Rehaklinik, die nephrologische Patienten nach Transplantation behandelt, konzeptionell, strukturell und personell auf die Behandlung dieser komplex kranken Patienten eingerichtet sein [14], damit der Patient von der Rehamaßnahme auch profitiert und nicht durch mangelnde Expertise des Behandlerteams gefährdet wird. Zielführend sind die Leitung der Klinik durch einen Nephrologen (mit Erfahrung in Transplantationsmedizin), eine fachspezifische Rufbereitschaft und eine fachspezifische Kompetenz des gesamten Therapeutenteams. Eine Dialysemöglichkeit muss ebenfalls vorhanden sein.

Schließlich müssen der Patient, sein behandelnder Nephrologe, das Transplantationszentrum und der Kostenträger davon überzeugt sein, dass der Patient von einer Rehamaßnahme selbst in einer spezialisierten Rehaklinik auch profitiert. Hier wird der Ruf nach klaren Daten laut [26]. Die Studienlage zum Erfolg von stationären Rehamaßnahmen bei nephrologischen Patienten ist auch aus oben genannten Gründen dürftig [15]. Es gibt jedoch klare Evidenz, dass bei nephrologischen und insbesondere bei transplantierten Patienten eine intensive Nachsorge zu einem besseren Verlauf der Erkrankung beiträgt [17], dass z. B. Schulungen bei nephrologischen Patienten mit einem besseren Outcome korreliert sind [22] und dass diese Patienten eine klare Unterstützung benötigen, um regelmäßige körperliche Aktivität in ihren Alltag zu integrieren [6]. Eine reine Fokussierung auf einzelne Parameter, wie z. B. den Kreatininverlauf, wird unseres Erachtens der Komplexität eines nephrologischen bzw. transplantierten Patienten nicht gerecht.

Wir sind davon überzeugt, dass Patienten nach Transplantation und nach Lebendspende von einer multimodalen multidisziplinären Rehabilitation in Kooperation mit den Einweisern bei den vielfältigen Anforderungen direkt nach den Eingriffen und im Langzeitverlauf profitieren. Die Komplexität der Beeinträchtigungen nierenkranker Patienten macht es kaum möglich, die beschriebenen Rehabilitationsmaßnahmen mit nephrologischer Hauptindikation im ambulanten Setting zielführend durchzuführen.

## Ausblick

Um die Studienlage zur Reha nephrologischer Patienten zu verbessern, wurde jüngst ein in Deutschland einzigartiges Projekt für Wartelistenpatienten initiiert, das vom Bayerischen Staatsministerium für Gesundheit und Pflege und der Arbeitsgemeinschaft der Krankenkassenverbände in Bayern unterstützt und vom Transplantationszentrum der Universität Erlangen, von der Fachklinik Bad Heilbrunn und von niedergelassenen Nephrologen im Raum Erlangen-Nürnberg durchgeführt wird [24]. Hierbei nehmen Dialysepatienten auf der Warteliste an einer strukturierten stationären Rehabilitation teil und werden dann ambulant vom Transplantationszentrum und von den niedergelassenen Nephrologen, ausgehend von dem in der Reha Erarbeiteten, auf verschiedenen Ebenen weiterbetreut. Über einen längeren Zeitraum werden regelmäßig im Studienprotokoll aufgelistete Assessments erhoben. Falls sich ein Patient auf der Warteliste verschlechtert, soll eine erneute Reha ihn wieder „fit für die Nierentransplantation“ machen. Ziel der Studie ist es zu zeigen, dass intensive multimodale Vorsorge inkl. Rehabilitation auf der Warteliste [7] zu einem besseren Outcome nach Transplantation führt.

Es wäre wünschenswert, wenn in Zukunft ähnliche kontrollierte Studien auch für transplantierte Patienten und Lebendspender durchgeführt werden würden.

## Fazit für die Praxis

- Eine stationäre multimodale Rehamaßnahme kann die ambulante Nachsorge nach Nierentransplantation und Nierenlebendspende nach dem Eingriff und im Langzeitverlauf sinnvoll ergänzen.
- Die Rehaklinik muss konzeptionell, strukturell und personell auf die Behandlung der komplexen nephrologischen und transplantierten Patienten eingerichtet sein.
- Rehaziele nach Transplantation: Steigerung von Lebensqualität, physische und psychische Stabilität, Sicherheit im Umgang mit dem Transplantat, Verringerung potenzieller Risikofaktoren für die Transplantatfunktion, Verbesserung sozialer und beruflicher Teilhabe.
- Rehaziele nach Nierenlebendspende: Nierenschutz, Verhinderung/Reduktion von physischen/psychischen Folgeschäden nach der Lebendspende, Einbeziehung des Kontextfaktors Empfänger zur Konsolidierung der neuen Situation.
- Die spezialisierte Rehamaßnahme setzt sich aus folgenden Therapiemodulen zusammen: medizinische Evaluation und Betreuung, psychologische Leistungen, Physio- und Sporttherapie, Schulungen, Sozialberatung.
